# The role of infant presence and other social correlates of fetal loss in wild mandrills

**DOI:** 10.1371/journal.pbio.3004034

**Published:** 2026-11-04

**Authors:** Alexander J. Pritchard

**Affiliations:** 1 Neuroscience and Behavior Unit, National Biomedical Research Institute, University of California Davis, Davis, California, United States of America; 2 Department of Population Health and Reproduction, Weill School of Veterinary Medicine, University of California Davis, Davis, California, United States of America

## Abstract

The survival of offspring is an important determinant of fitness. This Primer explores a new study in PLOS Biology examining how early conceiving female mandrills with an infant have a higher probability of fetal loss, relative to other females.

## Teaser

The survival of offspring is an important determinant of fitness. A new study in *PLOS Biology* examines how early conceiving female mandrills (*Mandrillus sphinx*) with an infant have a higher probability of fetal loss, relative to other females.

Group living and long developmental periods are concomitant with numerous complexities in social evolution. For instance, intraspecific competition can manifest in conflict between sexes or siblings, while the extended period of development for mammals with slower life histories has been posited to increase the likelihood for mother-offspring conflict [1–4]. These forms of competition and conflict are complex because they require us to consider fitness outcomes amid social pressures across varying degrees of relatedness and demographic states (Fig 1a). Accounting for these complexities that are intrinsic to the social environment remains essential to advance our knowledge of how animals negotiate their lifetime fitness strategies. This achievement is one of several from a new paper by Smit and colleagues [5], published in *PLOS Biology*. Their research examined fetal loss in wild mandrills (*Mandrillus sphinx*), a species of primate, to understanding the surrounding circumstances in a socially complex organism.

**Fig 1:**
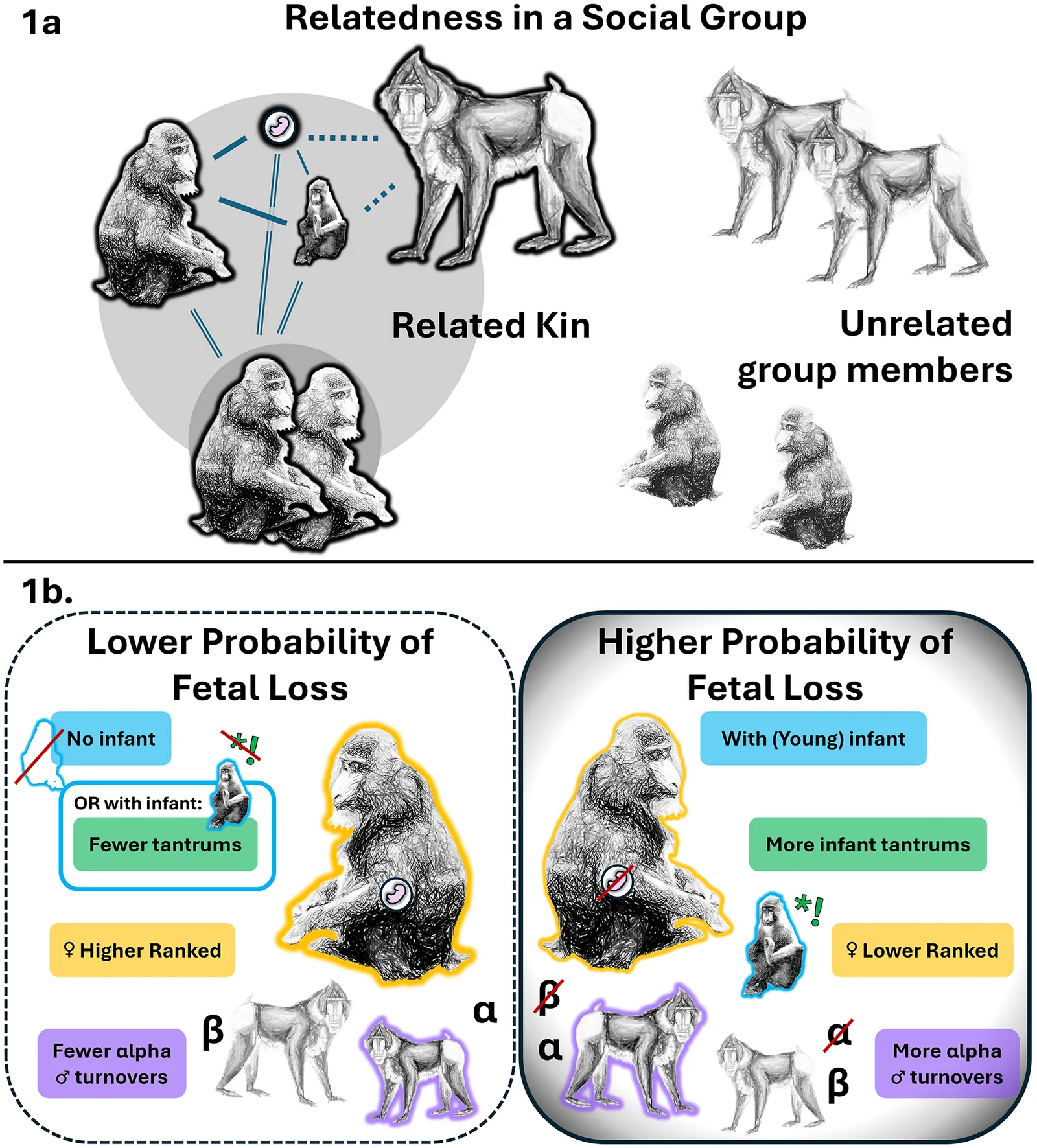
Kin structure and reproductive dynamics in social groups. **(A)** Relatedness and demographic state varies across group members. Here, we might envision high relatedness (connecting lines) between a mother, her offspring, and their matriline. Kin selection suggests that cooperation will be more common among related individuals, relative to unrelated individuals. Males might have uncertain paternal relatedness to offspring, which might mediate the strength of conflict between the sexes or parent-offspring (or, in some species, likelihood of infanticide [6]). **(B)** Major findings by Smit and colleagues [5] emphasize the associated role of sibling infants with fetal loss in mandrills (*Mandrillus sphinx*). This work highlights a potential source of variation that could alter females’ lifetime reproductive output. Photo credits: mandrill images are from the public domain.

One of the compelling aspects of this study was the scope of their work, which encompassed 11 years of data and 356 pregnancies, leveraging the capacity to noninvasively interpret pregnancy state through swellings [5]. Sexual swellings are an engorging of the anogenital region that often indicates receptivity but, in this instance, the anogenital region also cues pregnancy status. The authors demonstrate that some female mandrills conceive in consecutive mating seasons, which affords a more rapid reproductive strategy; yet, fetal loss emerges as a risk factor (Fig 1b). Fetal loss is especially relevant for primates due to their relatively low lifetime fecundity, and because most species (with a few exceptions) are typically uniparous—birthing one offspring at a time. Smit and colleagues [5] found that fetal loss was higher in mothers that currently had a young offspring, especially if it was less than 1 year of age. Pregnant mothers were more likely to reject their current infant by physical aggression, relative to nonpregnant mothers. Additionally, tantrum behaviors of current infants were associated with fetal loss. Together, these lines of evidence were used to substantiate the concept that the interplay between a pregnant mother and her current infant was associated with the loss of a fetus. Smit and colleagues [5] posit that, in instances where females are unable to sustain both infant ‘demand’ and fetal development, fetal loss mitigates against any further costs that would be accrued should the fetus come to term and then die.

This work provides an exciting opportunity to consider fundamental questions regarding how we critically examine social evolution through conflict, competition, and cooperation. The interplay between the infant and the fetus was of particular interest, as the offspring are competing for access to maternal resources yet cannot directly interact. Therefore, this competition is constrained to occurring through maternal-offspring conflict, though this process of competition would be expected to occur in tandem to ‘direct’ maternal-offspring conflict that both the infant and fetus would also, independently, be party to. The fetus and infant, despite being distinct cohorts, are concurrently drawing from a finite pool of maternal resources as might be expected for within brood competition [7], rather than impeding future (unrealized) reproductive potential of the mother. Though the infant and fetus are aligned in securing higher present reproductive investment, which can be at odds with future (unrealized) reproductive potential for the mother. At least one outcome of this conflict is clear, however, in that Smit and colleagues [5] demonstrate the capacity for females to recoup reproductive potential faster with the loss of the fetus, relative to the offspring’s loss once it reaches infancy.

Importantly, sustaining a young (<1 year) infant alongside a developing fetus occurs within a complex social environment such that there are many factors that could contribute to fetal loss. This point is salient because two additional social circumstances were associated with high probabilities of fetal loss: low social dominance rank, and a high incidence of alpha male turnovers (despite lack of support for male sexual intimidation). Both points emphasize stressors that have been previously associated with reproductive suppression but have not, to my knowledge, been brought into dialog with the current study’s focus on infant behavior. These concurrent processes are not necessarily independent and it remains an open question whether the effects are compounding or synergistic. For example, how are outcomes altered by infant relatedness to the alpha male? Does female dominance have direct energetic constraints that preferentially impede reproductive investment in the fetus, or infant? Are higher-ranked infants able to secure greater resources prior to their mother’s early conception? Even so, the findings by Smit and colleagues [5] might generally suggest that females prioritize their infants as the more independent reproductive output (relative to the fetus) when they are under multiple avenues of socio-ecological duress.

Maternal-offspring and sibling competition are important perspectives in investigating the adaptive strategies for how developing individuals leverage maternal resources. Such work informs how we understand social pressures, while also contributing to the generation of inquiries on broader scale group dynamics that introduce intervening pressures on resource access or limitations. Testing the mechanisms of fetal loss is an important next step of scientific inquiry [5], yet of equal importance would be to interrogate whether pathways of action are similar across these axes of social competition. For instance, in geladas (*Theropithecus gelada*), the rapidity of fetal loss after male takeovers was provided as evidence to suggest that geladas exhibited a distinct mechanism of fetal loss after takeovers, the Bruce effect, rather than chronic stress [8]. In this study [5], multiple social processes were associated with fetal loss such that it is pertinent to interrogate whether there is a singular pathway of generalized stress whereby reproductive suppression is attributable to allostatic load [8]. Such inquiries are necessary to understand proximate and ultimate causation, as well as whether distinct social dynamics act using independent mechanistic pathways.

Understanding how the reproductive strategy described by Smit and colleagues [5] intersects with sexual conflict theory, specifically on strategies and counterstrategies of infanticide, is an additional and compelling research direction. Infanticide results in a benefit when unrelated males are able to bring the female back into reproductive receptivity earlier than if the infant were to continue its development [6]. Seasonal breeding, though, limits the benefit of infanticide if females do not return to estrus off-season. Yet mandrills exhibit such long interbirth intervals [5] that death of an unweaned infant might be expected to return a female to receptivity by the next breeding season—a quality advantageous to infanticide [6]. Clearly, however, at least a subset of females are receptive in consecutive breeding seasons even with an unweaned infant. There are numerous female counterstrategies to male infanticide, many of which are pertinent to consider here, including: the Bruce effect, the role of swellings, or copulations during lactation [6].

One final resounding point emerges from the observation that mandrills have percentages of fetal loss (14.6%) within the ranges from two other old world monkeys and humans [5]. This point resonates because it implies that the socio-environmental circumstances described in mandrills can inform how we construct future research on fetal loss in other species of primates, or social animals more broadly. Although fetal loss can be difficult to determine in other species, this work inspires a greater focus on understanding loss of reproductive investment—especially in animals with wide reproductive intervals.
